# Supplementary material for: Pathogenicity and Relative Abundance of Dickeya and Pectobacterium Species in Switzerland: An Epidemiological Dichotomy
Source: Microorganisms. 2021 Oct 31;9(11):2270. doi: 10.3390/microorganisms9112270 (PMC8624237; doi:10.3390/microorganisms9112270)
Supplement: Supplementary file 1 [file microorganisms-09-02270-s001.zip › microorganisms-1406042-supplementary.pdf]

Supplementary Material

Table S1. Meteorological data for a representative field in the center of the seed potato growing area (Düdingen, FR, Switzerland) from 2010 to 2021.

year	average monthly temperature (°C)				
	April	Mai	Juni	July	average
2010	9.3	11.4	15.9	19.8	*14.1*
2011	11.2	14.3	15.9	16.2	*14.4*
2012	7.7	13.1	16.8	17.5	*13.8*
2013	7.9	9.5	15.0	19.6	*13.0*
2014	10.0	11.5	17.2	17.0	*13.9*
2015	9.2	13.5	17.8	22.0	*15.6*
2016	7.9	11.8	15.9	19.1	*13.7*
2017	8.0	13.3	18.8	19.0	*14.8*
2018	11.6	14.0	17.6	20.0	*15.8*
2019	7.8	9.9	18.3	20.3	*14.1*
2020	11.1	13.1	15.6	19.4	*14.8*
2021	6.9	10.0	17.8	17.7	*13.1*

year	monthly precipitation (mm)				
	April	Mai	Juni	July	total
2010	31	114	83	61	*289*
2011	17	81	139	113	*350*
2012	112	29	117	121	*379*
2013	114	69	59	122	*364*
2014	106	153	84	221	*564*
2015	83	146	70	23	*323*
2016	160	135	198	167	*660*
2017	64	108	80	92	*344*
2018	27	53	110	58	*247*
2019	58	106	73	48	*286*
2020	30	73	141	9	*254*
2021	19	147	219	250	*635*
